# Deubiquitinase USP2 stabilizes the MRE11–RAD50–NBS1 complex at DNA double-strand break sites by counteracting the ubiquitination of NBS1

**DOI:** 10.1016/j.jbc.2022.102752

**Published:** 2022-11-25

**Authors:** Hyunsup Kim, Dongmin Kim, Hyemin Choi, Gwangsu Shin, Joon-Kyu Lee

**Affiliations:** 1 Interdisciplinary Graduate Program in Genetic Engineering, Seoul National University, Seoul, Korea; 2 Department of Biology Education, Seoul National University, Seoul, Korea

**Keywords:** USP2, the MRE11–RAD50–NBS1 complex, DNA double-strand break, NBS1, ubiquitination, DSB, double-strand break, FBS, fetal bovine serum, HR, homologous recombination, NCS, neocarzinostatin

## Abstract

The MRE11–RAD50–NBS1 (MRN) complex plays essential roles in the cellular response to DNA double-strand breaks (DSBs), which are the most cytotoxic DNA lesions, and is a target of various modifications and controls. Recently, lysine 48-linked ubiquitination of NBS1, resulting in premature disassembly of the MRN complex from DSB sites, was observed in cells lacking RECQL4 helicase activity. However, the role and control of this ubiquitination during the DSB response in cells with intact RECQL4 remain unknown. Here, we showed that USP2 counteracts this ubiquitination and stabilizes the MRN complex during the DSB response. By screening deubiquitinases that increase the stability of the MRN complex in RECQL4-deficient cells, USP2 was identified as a new deubiquitinase that acts at DSB sites to counteract NBS1 ubiquitination. We determined that USP2 is recruited to DSB sites in a manner dependent on ATM, a major checkpoint kinase against DSBs, and stably interacts with NBS1 and RECQL4 in immunoprecipitation experiments. Phosphorylation of two critical residues in the N terminus of USP2 by ATM is required for its recruitment to DSBs and its interaction with RECQL4. While inactivation of USP2 alone does not substantially influence the DSB response, we found that inactivation of USP2 and USP28, another deubiquitinase influencing NBS1 ubiquitination, results in premature disassembly of the MRN complex from DSB sites as well as defects in ATM activation and homologous recombination repair abilities. These results suggest that deubiquitinases counteracting NBS1 ubiquitination are essential for the stable maintenance of the MRN complex and proper cellular response to DSBs.

DNA double-strand breaks (DSBs) are the most cytotoxic DNA lesions that induce genome instability, resulting in carcinogenesis, genetic diseases, and cell death (1, 2). To maintain genome integrity against DSBs, cells have evolved to activate checkpoint signaling for cell cycle control and repair DSBs *via* two major repair pathways nonhomologous end joining and homologous recombination (HR) (3). Nonhomologous end joining, an error-prone repair process, occurs throughout the cell cycle, whereas HR is an error-free repair pathway that functions in the S/G2 phase (4). In these cellular responses to DSBs, various proteins are recruited to DSB sites to act as sensors, signal transducers, or effectors, which are coordinated by PI3Ks, such as ataxia telangiectasia-mutated (ATM) and DNA-dependent protein kinase (DNA-PK) (5, 6).

In the current model of DSB response, the MRE11–RAD50–NBS1 (MRN) complex plays key roles as a sensor and a signal transducer by rapidly recognizing and locating DSBs (7). After binding to DSB sites, the MRN complex recruits and activates ATM (8). Activated ATM phosphorylates many substrates, including histone H2AX and MDC1 (9, 10), which in turn recruit more MRN complexes and ATM to DSB sites and consequently amplify the MRN-ATM signaling (11, 12). The MRN complex also acts as an effector in the HR repair process by participating in end resection (13). This activity is promoted by the binding of CtBP-interacting protein (CtIP), a cofactor of the MRE11 endonuclease (14). After binding of CtIP to the MRN complex, short-range resection of the DSB ends is initiated (15), and further processing by EXO1 and DNA2 generates extensive 3′-end protruding single-strand DNA (16). This 3′-overhang is coated by single-strand DNA–binding protein, RPA, followed by replacement of RAD51 for homology search and DNA strand invasion (17).

As the MRN complex plays key roles in the DSB response from damage sensing to repair, it is one of the major control targets of various modifications such as phosphorylation and ubiquitination. ATM phosphorylates all three components of the MRN complex, which is critical for checkpoint activation and DSB repair (18). Phosphorylation of MRE11 by polo-like kinase 1 inhibits the recruitment of the MRN complex to DSBs (19). CDK phosphorylation of NBS1 influences the choice of repair pathway for dysfunctional telomeres (20). The ubiquitination-dependent control of the MRN complex has also been reported in many studies. Lysine (K) 6-linked ubiquitination of NBS1 proteins by the ring finger protein RNF8 promotes optimal binding to DSBs (21). K63-linked ubiquitination of NBS1 by SKP2 E3 ubiquitin ligase and Pellino1 facilitates ATM activation and binding of the MRN complex to DSB sites, respectively (22, 23).

Recently, premature disassembly of the MRN complex from DSB sites by SKP2-dependent ubiquitination of NBS1 was observed in cells lacking RECQL4 helicase activity and overexpression of a deubiquitinase, USP28, restored the stability of the MRN complex at DSB sites, ATM activation, and HR repair in these cells (24). As K48-linked ubiquitination of NBS1 by SKP2 E3 ligase also occurs in cells with intact RECQL4 helicase and overexpression of USP28 reduces the ubiquitination of NBS1, these results suggest that there may be a novel control mechanism that plays an important role in the DSB response by ubiquitination and deubiquitination of NBS1. However, the cellular role of the K48-linked ubiquitination and deubiquitination of NBS1 is still obscure because premature disassembly of the MRN complex during the DSB response was observed only in RECQL4-defective cells and depletion of USP28 did not markedly influence the DSB response, except in a few cell lines such as H460 (25).

In this study, we identified USP2 as a new deubiquitinase that acts at DSB sites to counteract NBS1 ubiquitination by SKP2 E3 ligase. Live-cell imaging of cells expressing EGFP-fused USP2 after laser microirradiation, mapping of domains responsible for recruitment to DSB sites, and analyses of phospho-deficient and phospho-mimetic mutants of USP2 showed that USP2 is recruited to DSB sites in a ATM-dependent manner, and phosphorylation of two critical residues in the N terminus of USP2 is essential for its recruitment to DSBs and interaction with RECQL4. By inactivation of USP2 and USP28, we also showed that the action of deubiquitinases counteracting NBS1 ubiquitination is essential for the stable maintenance of the MRN complex on DSB sites and proper cellular response to DSBs.

## Results

### Overexpression of USP2 restores the DSB response in RECQL4-defective cells

**Figure 1:**
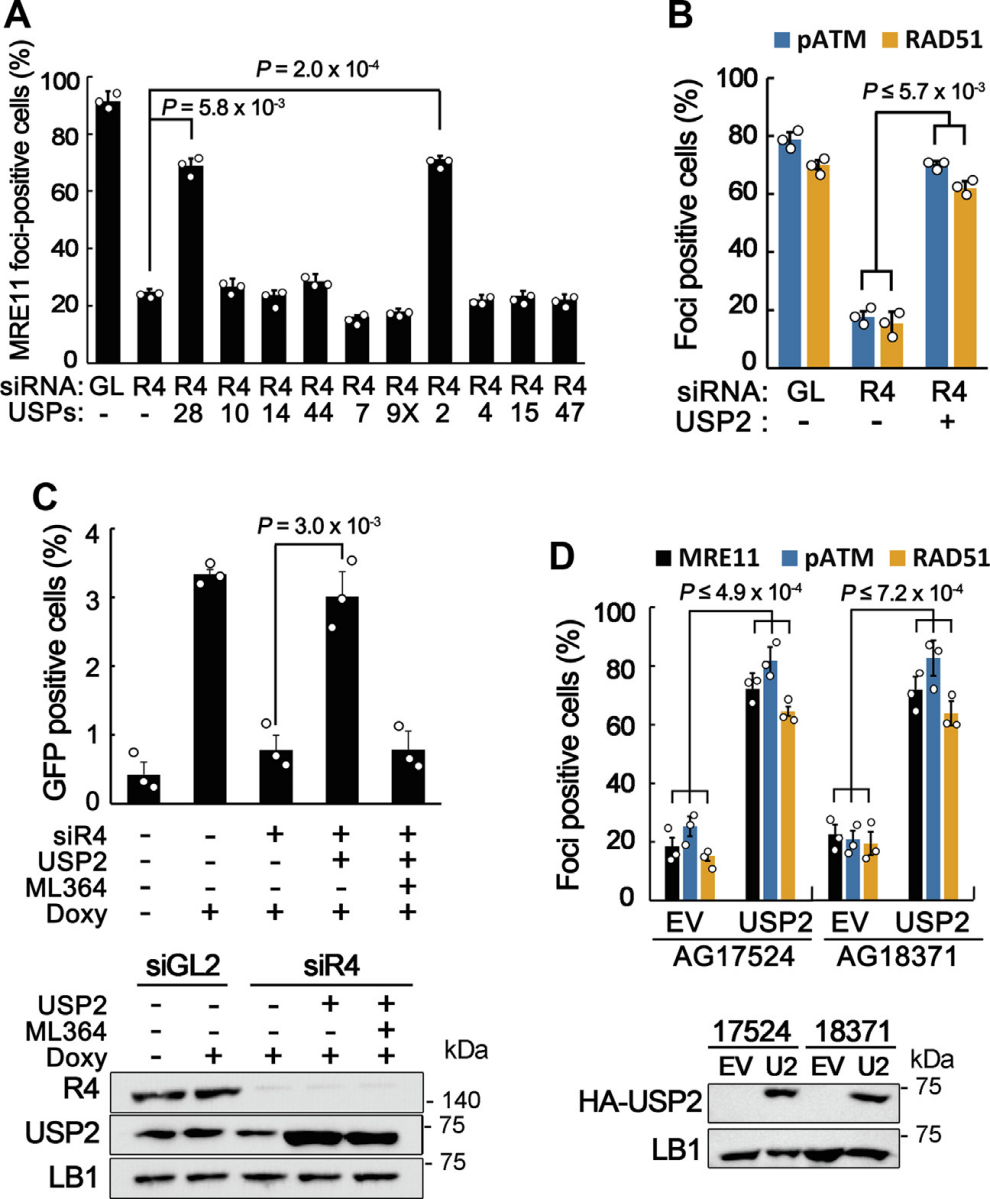
**Overexpression of USP2 restores the DSB response in RECQL4-defective cells.***A*, screening of deubiquitinases increasing stability of the MRN complex in RECQL4-depleted cells. U2OS cells transfected with the indicated siRNAs and plasmids were treated with NCS for 15 min and incubated in fresh medium for 1 h. MRE11 immunostaining was carried out and more than 50 cells were counted for foci number in each experiment. Data in graphs are means ± SD; n =3. R4, RECQL4. *B*, immunostaining of pATM and RAD51 in mock- (siGL) or RECQL4-depleted (siR4) U2OS cells transfected with empty or USP2 plasmids. Cells were treated with NCS and incubated in fresh medium for 1 h. Data in graphs are means ± SD; n = 3. *p* values for pATM, 4.0 × 10^−4^; RAD51, 5.7 × 10^−3^. *C*, HR repair assay using TRI-DR-U2OS cells. Mock- or RECQL4-depleted cells were transfected with empty or USP2 plasmids. After 24 h incubation, cells were treated with doxycycline to induce I-*Sce*1 and incubated for additional 48 h. For inhibiting USP2, 20 μM of ML364 was treated 1 h before doxycycline treatment. Data in graphs are means ± SD.; n =3. *Lower panel* is the Western blots showing depletion of RECQL4 and USP2 expression. *D*, immunostaining of MRE11, pATM, or RAD51 in Rothmund–Thomson syndrome cells (AG17524 and AG18371) transfected with empty (EV) or USP2 plasmids. Cells were treated with NCS and incubated in fresh medium for 1 h. Data in graphs are means ± SD; n = 3. *p* values for AG17524 MRE11, 4.9 × 10^−4^; pATM, 3.2 × 10^−4^; RAD51, 1.4 × 10^−5^; AG18371 MRE11, 4.1 × 10^−4^; pATM, 4.0 × 10^−4^; RAD51, 7.2 × 10^−4^. *Lower panel* is the Western blots showing expression of HA-tagged USP2. HR, homologous recombination; LB1, lamin B1; NCS, neocarzinostatin.

To explore the deubiquitinases that influence the stability of the MRN complex during the DSB response, we took advantage of RECQL4-depleted cells, in which the MRN complex is prematurely disassembled from DSB sites by ubiquitin-dependent degradation of the NBS1 protein (24). Several deubiquitinases that have been shown to be associated with DNA damage responses, cell cycle, or carcinogenesis were individually overexpressed in RECQL4-depleted cells, and MRE11 foci were examined after treating cells with neocarzinostatin (NCS), a DSB-inducing reagent. We found that overexpression of USP2 increased MRE11 foci-positive cells, as did USP28 overexpression, suggesting that premature disassembly of the MRN complex at DSB sites is prevented by USP2 (Fig. 1*A*). HR repair and ATM activation abilities, which were determined by the appearance of RAD51 and phospho-ATM (pATM) foci, respectively, were also restored by USP2 overexpression in RECQL4-depleted cells (Fig. 1*B*). The effect of USP2 overexpression on HR repair ability was further confirmed by an HR assay using DR-GFP HR reporter cells (TRI-DR-U2OS) (Fig. 1*C*). Restoration of MRE11, pATM, and RAD51 foci by USP2 overexpression was also observed in fibroblast cells collected from patients with Rothmund–Thomson syndrome, which is caused by mutations in the RECQL4 gene (Fig. 1*D*). Taken together, these results suggest that overexpression of USP2 prevented premature disassembly of the MRN complex from DSB sites and restored the DSB response in RECQL4-defective cells.

### USP2 is recruited to DSB sites and counteracts the ubiquitination of NBS1

**Figure 2:**
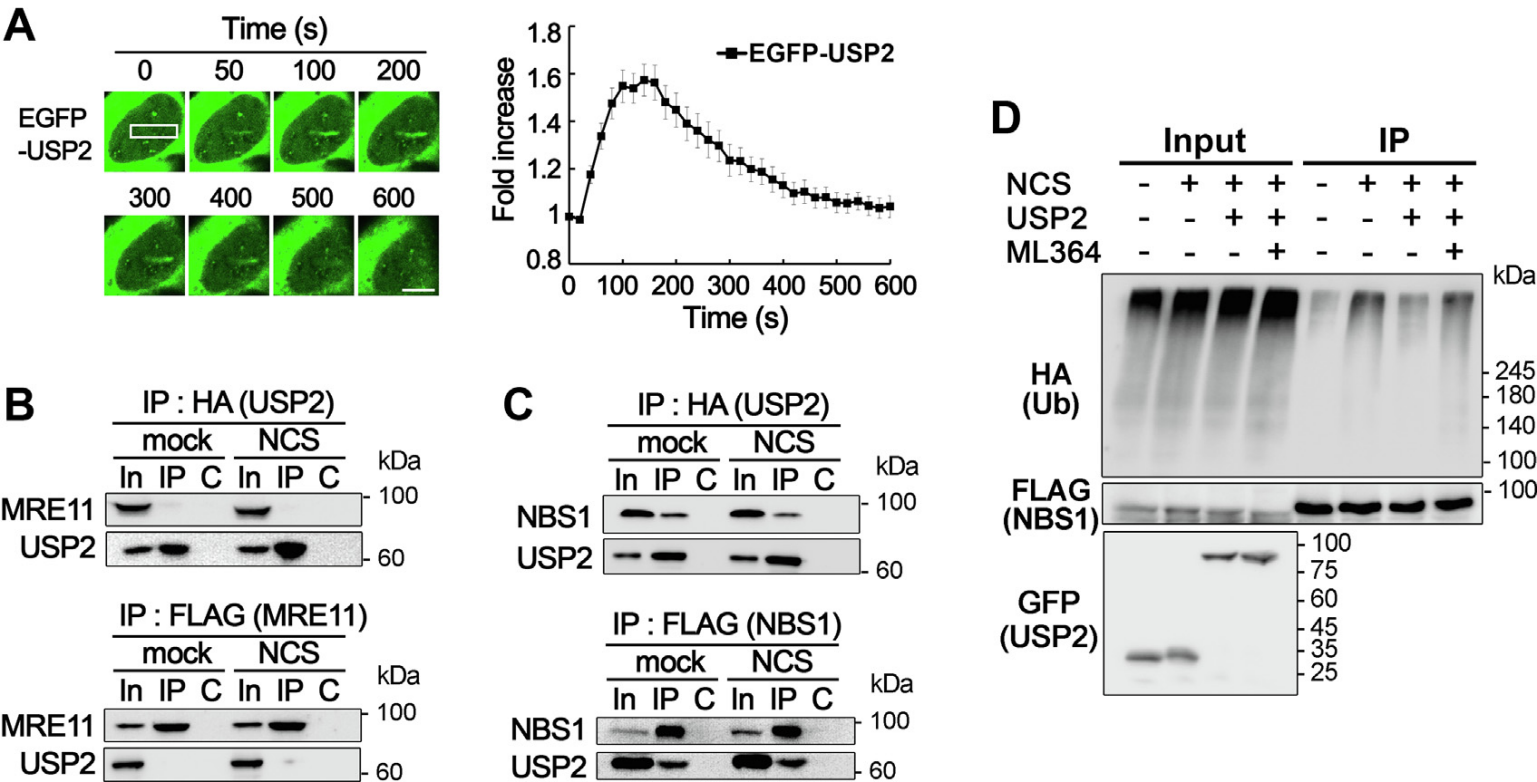
**USP2 is recruited to DSB sites and counteracts the ubiquitination of NBS1.***A*, EGFP-USP2 binding to microirradiation sites in U2OS cells. Data in graphs are means ± SEM; n = 20. The scale bar represents 10 μm. *B* and *C*, interaction of USP2 with MRE11 (*B*) or NBS1 (*C*) in mock- or NCS-treated U2OS cells. FLAG-tagged MRE11 or FLAG-tagged NBS1 was expressed with HA-tagged USP2 in U2OS cells, and immunoprecipitation (IP) was carried out with anti-HA antibodies (*upper panel*) or anti-FLAG-M2-agarose beads (*lower panel*). Anti-FLAG (for MRE11 or NBS1) and anti-HA (for USP2) antibodies were used for Western blotting. Lanes: In, 10% of input for IP; C, control IP with nonspecific IgG. *D*, ubiquitination of NBS1 in HEK293T cells treated as indicated. Cells transfected with HA-ubiquitin, FLAG-NBS1, and GFP-USP2 plasmids as indicated were pretreated with ML364 and MG132 1 h before NCS treatment. Anti-FLAG M2 agarose beads were used for IP, and anti-HA (for ubiquitin), anti-FLAG (for NBS1), and anti-GFP (for USP2) antibodies were used for Western blotting. DSB, double-strand break; HR, homologous recombination; NCS, neocarzinostatin.

Premature disassembly of the MRN complex from DSB sites has been shown to be caused by SKP2-dependent ubiquitination of the NBS1 protein in RECQL4-defective cells (24). To test the possibility that USP2 increases the stability of the MRN complex at DSB sites by counteracting the ubiquitination of NBS1, we examined the recruitment of USP2 to DSB sites. While EGFP-fused USP2 proteins expressed in U2OS cells were mostly located in the cytoplasm, small amounts of these proteins appeared in the nucleus and were rapidly bound to laser microirradiation sites (Fig. 2*A*). The level of protein binding peaked at approximately 150 s and then gradually decreased until 600 s. This result indicates that USP2 plays a role in the DSB response by directly acting on the targets at DSB sites. Coimmunoprecipitation analysis of U2OS cells overexpressing USP2 and MRE11 or USP2 and NBS1 indicated that USP2 barely interacted with MRE11 in the absence or presence of DNA damage (Fig. 2*B*). On the other hand, USP2 stably interacted with NBS1 upon NCS treatment, and their interaction persisted regardless of NCS treatment (Fig. 2*C*). We then examined whether USP2 influences DSB-dependent ubiquitination of NBS1 proteins. As shown in a previous study (24), ubiquitination of NBS1 was significantly increased by treatment with NCS, and this was reversed by USP2 overexpression to almost the same level as that in nondamaged cells (Fig. 2*D*). Treatment with ML364, a specific inhibitor of USP2 deubiquitinase, masked the effect of USP2 overexpression, indicating that increased activity of USP2 is responsible for the decrease in the ubiquitination of NBS1 (Fig. 2*D*). Taken together, these results suggest that USP2 prevents premature disassembly of the MRN complex in RECQL4-defective cells by counteracting the ubiquitination of NBS1.

### USP2 is recruited to DSB sites in a manner dependent upon ATM, PARP, and RECQL4

**Figure 3:**
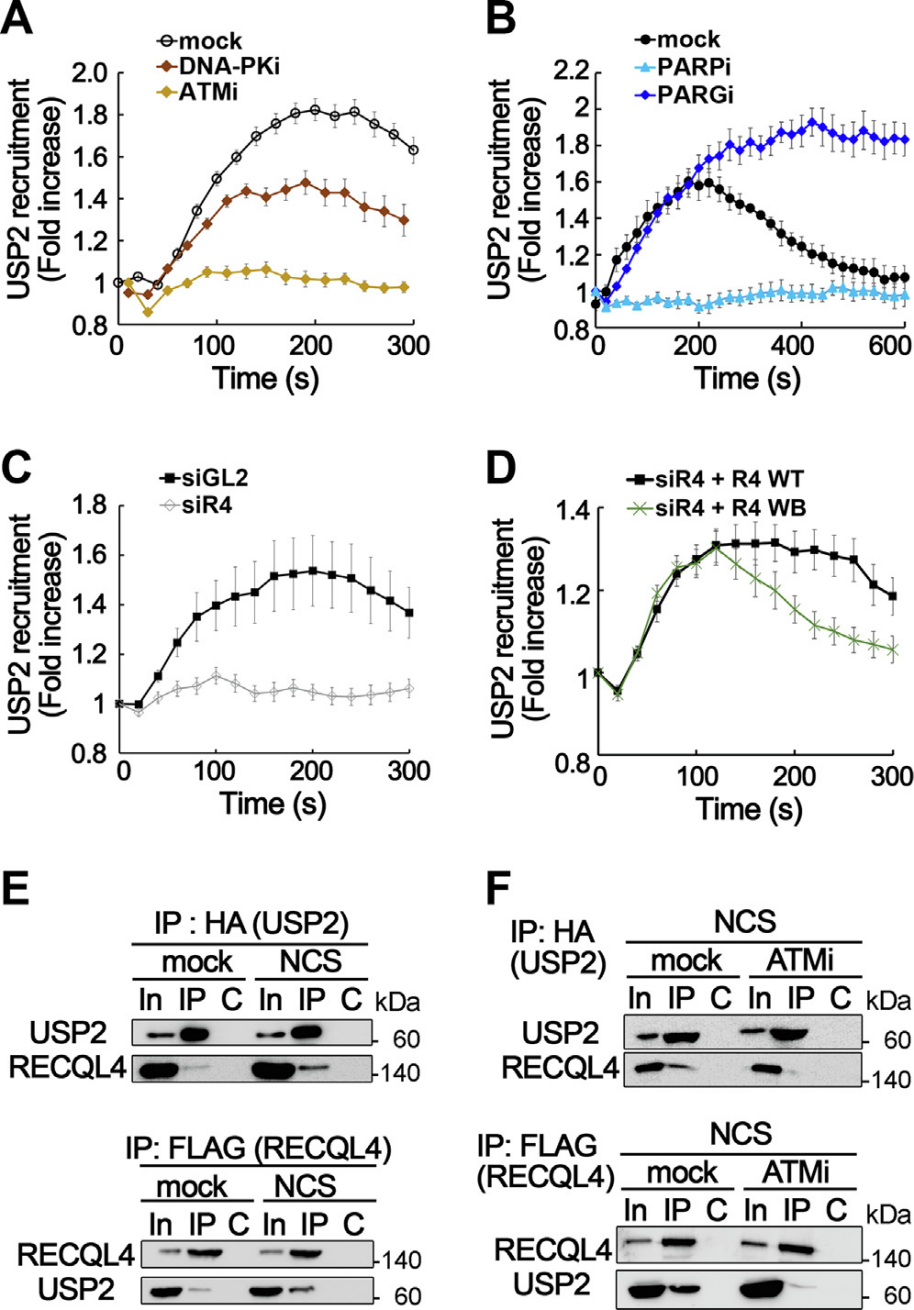
**USP2 is recruited to DSB sites in a manner dependent on ATM, PARP, and RECQL4.***A* and *B*, recruitment of EGFP-USP2 to laser microirradiation sites was analyzed in U2OS cells treated with inhibitors (20 μM) of ATM (KU55933), DNA-PK (NU7441), PARP (olaparib), or PARG (PDD00017273) 1 h before microirradiation. Data in graphs are means ± SEM.; n = 20. *C*, recruitment of USP2 to microirradiation sites in mock- (siGL) or RECQL4-depleted (siR4) U2OS cells. Data in graphs are means ± SEM; n = 20. *D*, RECQL4-depleted U2OS cells were transfected with WT or Walker B mutant (WB) RECQL4 plasmids and used for analyzing USP2 binding to microirradiation sites. Data in graphs are means ± SEM; n = 20. *E* and *F*, interaction between USP2 and RECQL4 in U2OS cells. U2OS cells transfected with FLAG-RECQL4 and HA-USP2 plasmids were used for IP with anti-HA antibodies (*upper panels*) or anti-FLAG-M2-agarose beads (*lower panels*). Cells treated with NCS (*E*) or NCS and ATM inhibitor (KU55933, 20 μM) (*F*) were used for IP. Anti-FLAG (for RECQL4) and anti-HA (for USP2) antibodies were used for Western blotting. Lanes: In, 10% of input for IP; C, control IP with nonspecific IgG. DSB, double-strand break; IP, immunoprecipitation.

As USP2 can act at DSB sites, we examined how its recruitment to DSB sites was controlled during the DSB response. In the DSB response, ATM and DNA-PK play important roles to initiate signaling cascade and to recruit many factors to DSB sites by phosphorylating various target proteins (26). To determine the role of these kinases in the recruitment of USP2, specific inhibitors of ATM and DNA-PK, KU55933 and NU7441, respectively, were individually treated in cells expressing EGFP-USP2 proteins, and recruitment of USP2 to the laser microirradiation site was analyzed by live-cell imaging. Treatment with the DNA-PK inhibitor decreased the level of USP2 binding to the microirradiation site but substantial USP2 binding was still observed in these cells (Fig. 3*A*). In contrast, treatment with the ATM inhibitor almost completely reduced USP2 binding to the microirradiation site (Fig. 3*A*), suggesting that ATM kinase activity is required for recruitment of USP2 to DSB sites. Another important player in the DSB response is poly(ADP-ribosyl)ation (PARylation), which is produced by poly(ADP-ribose) polymerases (PARPs) and removed by poly(ADP-ribose) glycohydrolase (PARG). PARylation also appeared to be critical for the recruitment of USP2 to DSB sites because treatment with olaparib, an inhibitor of PARP1 and PARP2, almost completely abolished the binding of USP2 to laser-induced DSB sites and treatment with the PARG inhibitor, PDD00017273, prevented dissociation of USP2 from DSB sites (Fig. 3*B*). Furthermore, depletion of RECQL4 abolished the recruitment of USP2 to laser-induced DSB sites (Figs. 3*C* and S1). The expression of helicase-defective RECQL4 mutant proteins (D605A and E606A in the Walker B motif) as well as WT RECQL4 proteins restored USP2 binding to laser-induced DSB sites in RECQL4-depleted cells. Therefore, physical presence of RecQL4, instead of its helicase activity, appeared to be required for the recruitment of USP2 to DSB sites (Figs. 3*D* and S1). However, dissociation of USP2 from DSB sites was faster in Walker B mutant-expressing cells than in cells with WT RECQL4 (Fig. 3*D*), suggesting that the helicase activity of RECQL4 may play a role in the maintenance of USP2 at DSB sites. Taken together, these results indicate that recruitment of USP2 to DSB sites depends on RECQL4 protein, PARylation, and ATM kinase activity. Interestingly, recruitment of RECQL4 proteins to DSB sites also requires PARylation (27). Therefore, the PARylation dependency of USP2 recruitment to DSB sites might be caused by the PARylation dependency of RECQL4 recruitment to DSB sites. To support this notion, USP2 interacted with RECQL4 in coimmunoprecipitation experiments and NCS treatment significantly increased this interaction (Fig. 3*E*). Furthermore, inhibition of ATM kinase activity almost completely abolished this interaction in NCS-treated cells (Fig. 3*F*). Taken together, these results suggest that USP2 may be recruited to DSB sites *via* its interaction with RECQL4, which is dependent on ATM kinase activity.

### The N terminus of USP2 is sufficient for ATM-dependent recruitment to DSB sites

**Figure 4:**
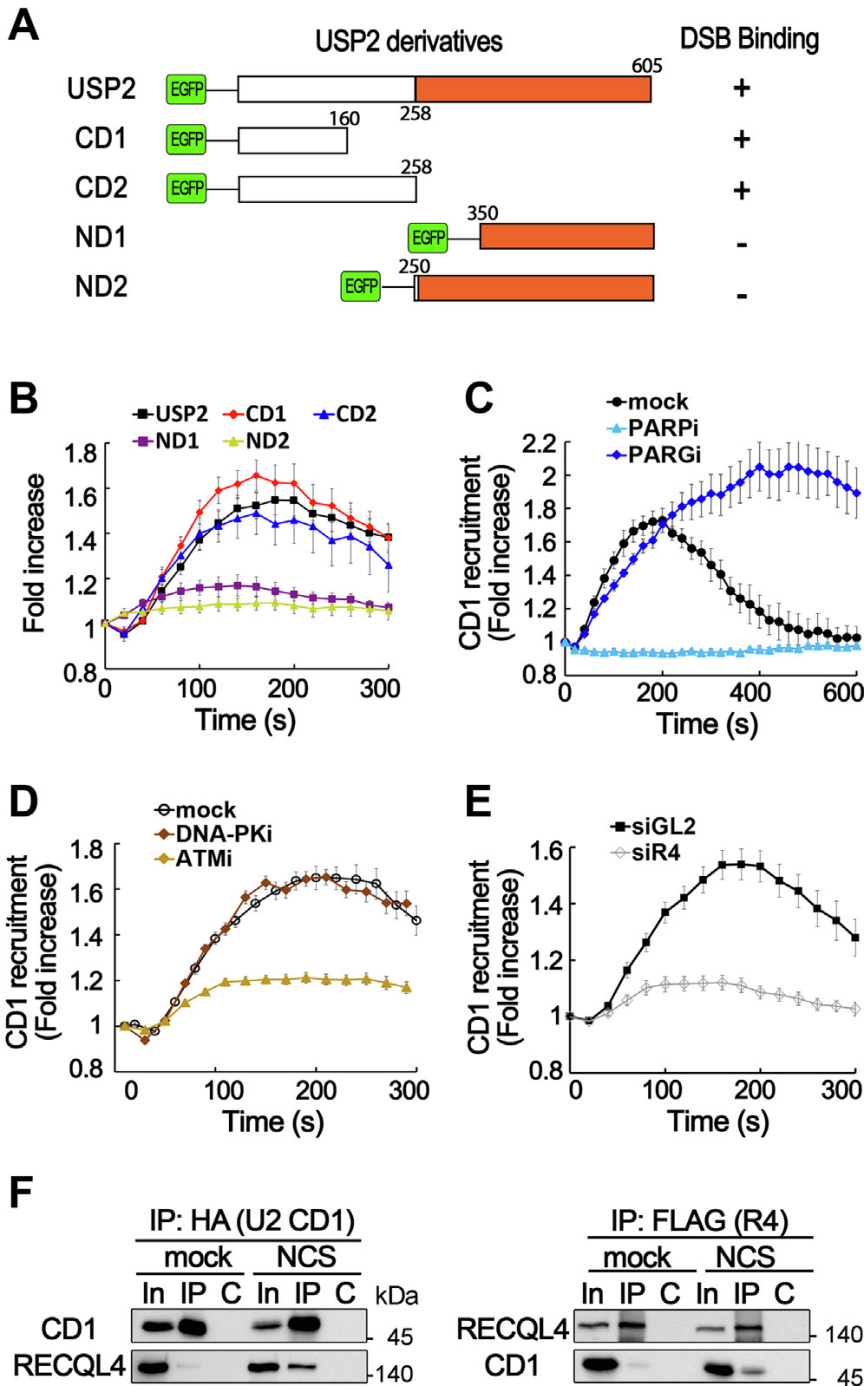
**USP2 binds to DSB sites through its N terminus.***A*, schematic diagram of truncated USP2 proteins and their binding to laser-induced DSB sites. The USP2 C terminus (*orange*) indicates conserved USP domain. *B*, EGFP-fused USP2 derivatives were expressed in U2OS cells and their binding to laser microirradiation sites were analyzed. Data in graphs are means ± SEM; n = 20. *C*–*E*, influences of PARylation (*C*), inhibition of DSB-inducing kinases (*D*), and RECQL4 depletion (*E*) on the recruitment of EGFP-fused CD1 to DSB sites. Inhibitors were treated 1 h before microirradiation. Data in graphs are means ± SEM; n = 20. *F*, interaction between HA-CD1 and FLAG-RECQL4 in mock- or NCS-treated U2OS cells. IP was carried out with anti-HA antibodies (*left panel*) or anti-FLAG-M2-agarose beads (*right panel*). Anti-FLAG (for RECQL4) and anti-HA (for CD1) antibodies were used for Western blotting. R4, RECQL4. Lanes: In, 10% of input for IP; C, control IP with nonspecific IgG. DSB, double-strand break; IP, immunoprecipitation.

To clarify the mechanism of USP2 recruitment to DSB sites, we determined the minimal USP2 region required for binding to DSB sites. For this purpose, several EGFP-fused truncated USP2 proteins were expressed (Fig. 4*A*) and their binding to the laser microirradiation site was examined by live-cell imaging. As shown in Figure 4, *A* and *B*, deletion of the USP domain at the C terminus did not significantly affect the binding to laser-induced DSB sites. In contrast, truncated proteins without N terminus were unable to bind to laser-induced DSB sites, and the N terminus of USP2 containing amino acid residues 1 to 160 (CD1) was sufficient to bind to DSB sites (Fig. 4, *A* and *B*). Similar to the full-length USP2 protein, this minimal region of USP2 bound to laser-induced DSB sites in a manner dependent on PARylation, ATM kinase activity, and RECQL4 (Fig. 4, *C*–*E*), suggesting that this region of USP2 contains all the requirements for recruitment to DSB sites. As this minimal region for DSB binding also stably interacted with RECQL4 in a DSB-dependent manner (Fig. 4*F*), USP2 might be recruited to DSB sites *via* interaction with RECQL4.

### Phosphorylation of two critical residues in USP2 N terminus is required for its recruitment to DSB sites and interaction with RECQL4

**Figure 5:**
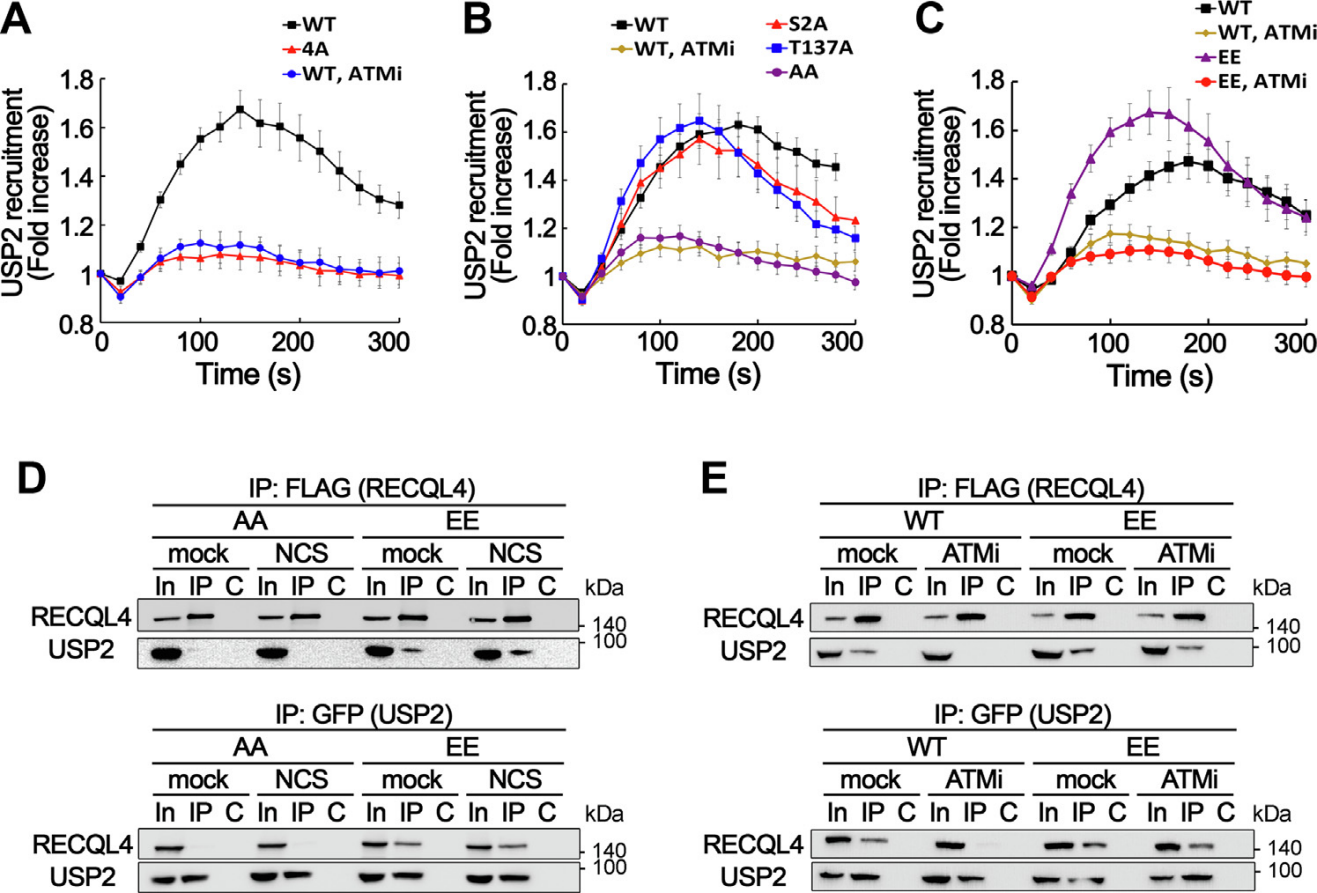
**Phosphorylation of two critical residues in USP2 N terminus by ATM is required for recruitment to DSB sites and interaction with RECQL4.***A*–*C*, recruitments of various alanine-substituted or glutamic acid–substituted full-length USP2 proteins to DSB sites were examined by laser microirradiation followed by live-cell imaging. ATM inhibitor (ATMi), KU55933, was treated 1 h before microirradiation. WT USP2; 4A, USP2 with substitution of four ATM phosphorylation sites (S2, S94, T137, S142) to alanine; AA, substitution of S2 and T137 to alanine; EE, substitution of S2 and T137 to glutamic acid. Data in graphs are means ± SEM; n = 20. *D* and *E*, interaction of phospho-deficient or mimetic mutant of USP2 with RECQL4. FLAG-RECQL4 and EGFP-USP2 mutant proteins were expressed in U2OS cells and treated as indicated. IP was carried out with anti-FLAG-M2-agarose beads (*upper panel*) or anti-GFP antibodies (*lower panel*). Anti-FLAG (for RECQL4) and anti-GFP (for CD1) antibodies were used for Western blotting. Lanes: In, 10% of input for IP; C, control IP with nonspecific IgG. DSB, double-strand break; IP, immunoprecipitation.

As the N-terminal domain of USP2 showed ATM-dependent recruitment to DSB sites, we further investigated whether phosphorylation of USP2 N terminus is important for its recruitment to DSB sites. The minimal region of the USP2 N terminus for DSB binding contains four SQ/TQ sites (S2, S94, T137, S142) that can be recognized and phosphorylated by ATM kinase. Therefore, we generated USP2 proteins with various combinations of alanine substitutions in these four S/T residues and determined their binding to laser-induced DSB sites. While individual alanine substitutions of serine or threonine in these putative phosphorylation sites did not significantly affect the recruitment of USP2 to DSB sites, alanine substitution of all four serine and threonine residues (4A) resulted in complete loss of DSB-binding activity (Fig. 5*A*). Furthermore, alanine substitution of two amino acid residues, S2A and T137A (AA), also led to the loss of DSB-binding activity (Fig. 5*B*), suggesting that phosphorylation of these two residues is critical for the recruitment of USP2 to DSB sites. The phospho-mimetic mutant USP2 with glutamic acid substitution at these two phosphorylation sites, S2E and T137E (EE), was able to bind to DSB sites, but its binding was disturbed by ATM inhibition (Fig. 5*C*), suggesting that the phosphorylation of these residues is required but not sufficient for recruitment to DSB sites. We then examined whether the phosphorylation of USP2 influences its interaction with RECQL4. As shown in Figure 5*D*, interaction between phospho-deficient USP2 (AA) and RECQL4 was not observed in the coimmunoprecipitation analysis. In contrast, phosphomimetic USP2 (EE) proteins stably interacted with RECQL4, both in the absence and presence of DNA damage (Fig. 5*D*), and inhibition of ATM kinase activity did not disrupt their interaction, although the interaction appeared to be slightly weak (Fig. 5*E*). Taken together, these results suggest that interaction of USP2 with RECQL4 through ATM-dependent phosphorylation of these two sites is not sufficient for the recruitment of USP2 to DSB sites. As the mutant USP2 with phosphomimetic substitution of all four ATM phosphorylation sites in the N terminus (4E) still showed ATM dependency in the recruitment to DSB sites (Fig. S2), phosphorylation of other proteins by ATM might also play an important role in the recruitment of USP2 to DSB sites. Collectively, these data suggest that ATM is a key regulator for USP2 recruitment to DSB sites by phosphorylating USP2 at S2 and T137, which is essential for the interaction between USP2 and RECQL4.

### USP2 and USP28 play a redundant role in the stable maintenance of the MRN complex at DSB sites during the DSB response

**Figure 6:**
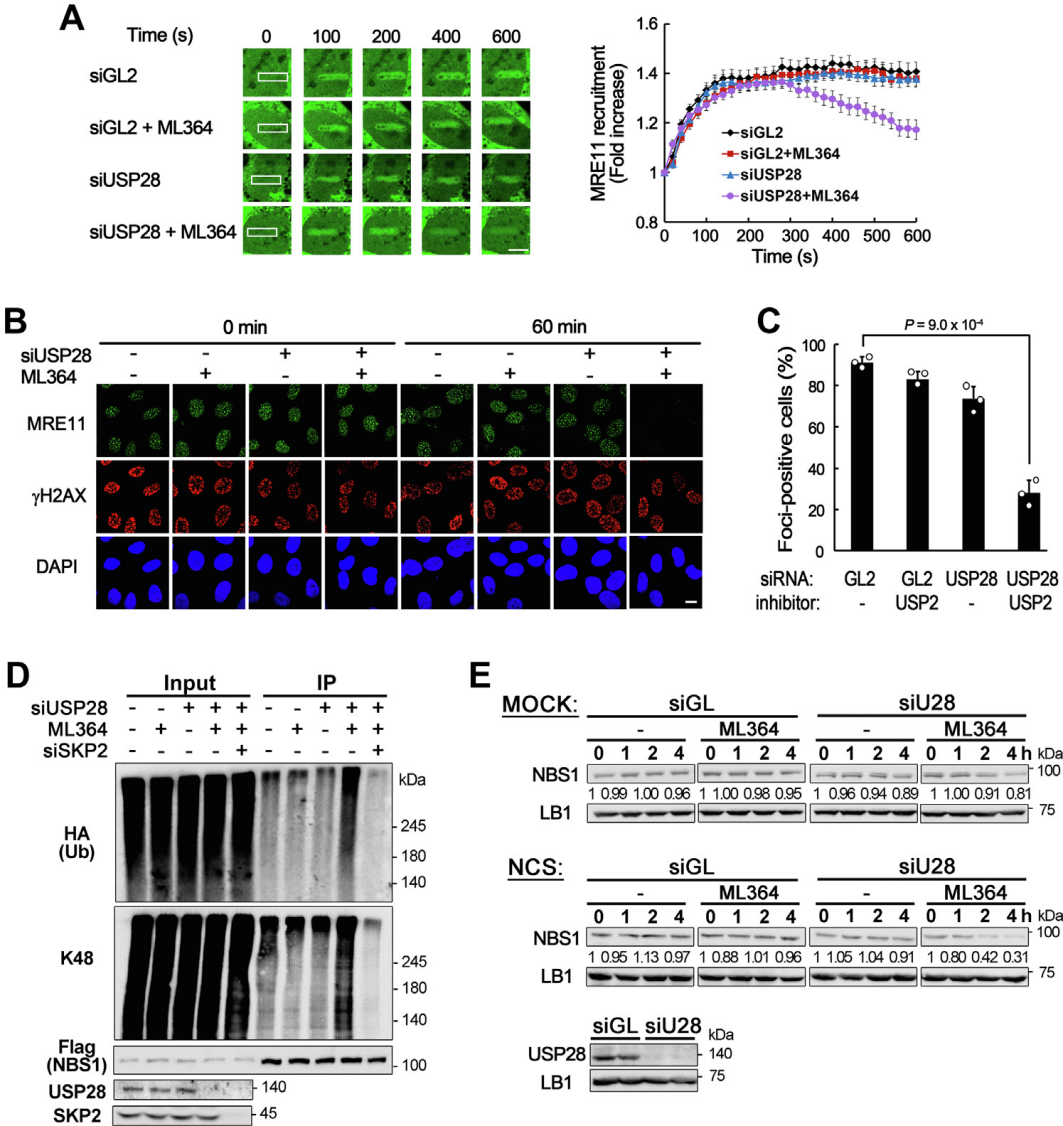
**Inactivation of both USP2 and USP28 activities results in premature disassembly of the MRN complex from DSB sites by increasing the ubiquitination of NBS1.***A*, binding of EGFP-MRE11 to microirradiation induced DSB sites in USP2 and/or USP28 inactivated cells. Cells transfected with mock (GL) or USP28 siRNAs were treated with USP2 inhibitor (ML364, 20 μM) 1 h before microirradiation. Representative images (*left panel*) and graph for relative green fluorescence intensity in damaged area (*right panel*) are shown. The scale bar represents 10 μm. Data in graphs are means ± SEM.; n = 20. *B* and *C*, immunostaining of MRE11 in USP2 and/or USP28 inactivated U2OS cells. Cells were transfected with the indicated siRNAs and/or treated with USP2 inhibitor (ML364, 20 μM) followed by NCS treatment and incubation in fresh medium for the indicated time. Representative images (*B*) and graph (*C*) for the percentage of MRE11 foci positive cells with 1 h incubation after NCS treatment. The scale bar represents 10 μm. Data in graphs are means ± SD; n = 3. *D*, ubiquitination of NBS1 in HEK293T cells transfected with indicated siRNAs and treated with USP2 inhibitor (ML364, 20 μM). Anti-FLAG M2 agarose beads were used for IP, and anti-K48 ubiquitin, anti-HA (for ubiquitin), anti-FLAG (for NBS1), and anti-USP28, anti-SKP2 antibodies were used for Western blotting. *E*, stability of NBS1 proteins in USP28-depleted and/or USP2 inhibited U2OS cells. Cells treated with NCS were incubated for the indicated time in the presence of cycloheximide (50 μg/ml) and Western blotting was carried out to determine the level of NBS1 protein. The levels of NBS1 proteins normalized to that of lamin B1(LB) are shown below each lane. DSB, double-strand break; IP, immunoprecipitation; NCS, neocarzinostatin.

As USP2 counteracted the ubiquitination of NBS1 to increase the stability of the MRN complex in RECQL4-defective cells, we examined whether USP2 plays a role in the DSB response in cells with intact RECQL4 helicase. For this purpose, U2OS cells were treated with ML364 to inhibit the deubiquitinase activity of USP2 and the stability of the MRN complex was examined after NCS treatment. As the binding of MRE11 protein to DSB sites depends on complex formation with RAD50 and NBS1 (28, 29), MRE11 is a good marker for tracing the MRN complex at DSB sites. As shown in Figure 6, *A* and *B*, neither EGFP-fused MRE11 (EGFP-MRE11) recruitment to microirradiation site nor foci formation of MRE11 were significantly influenced by inhibition of USP2 deubiquitinase activity, suggesting that USP2 activity may not be essential for the stable maintenance of the MRN complex at DSB sites in cells with intact RECQL4.

**Figure 7:**
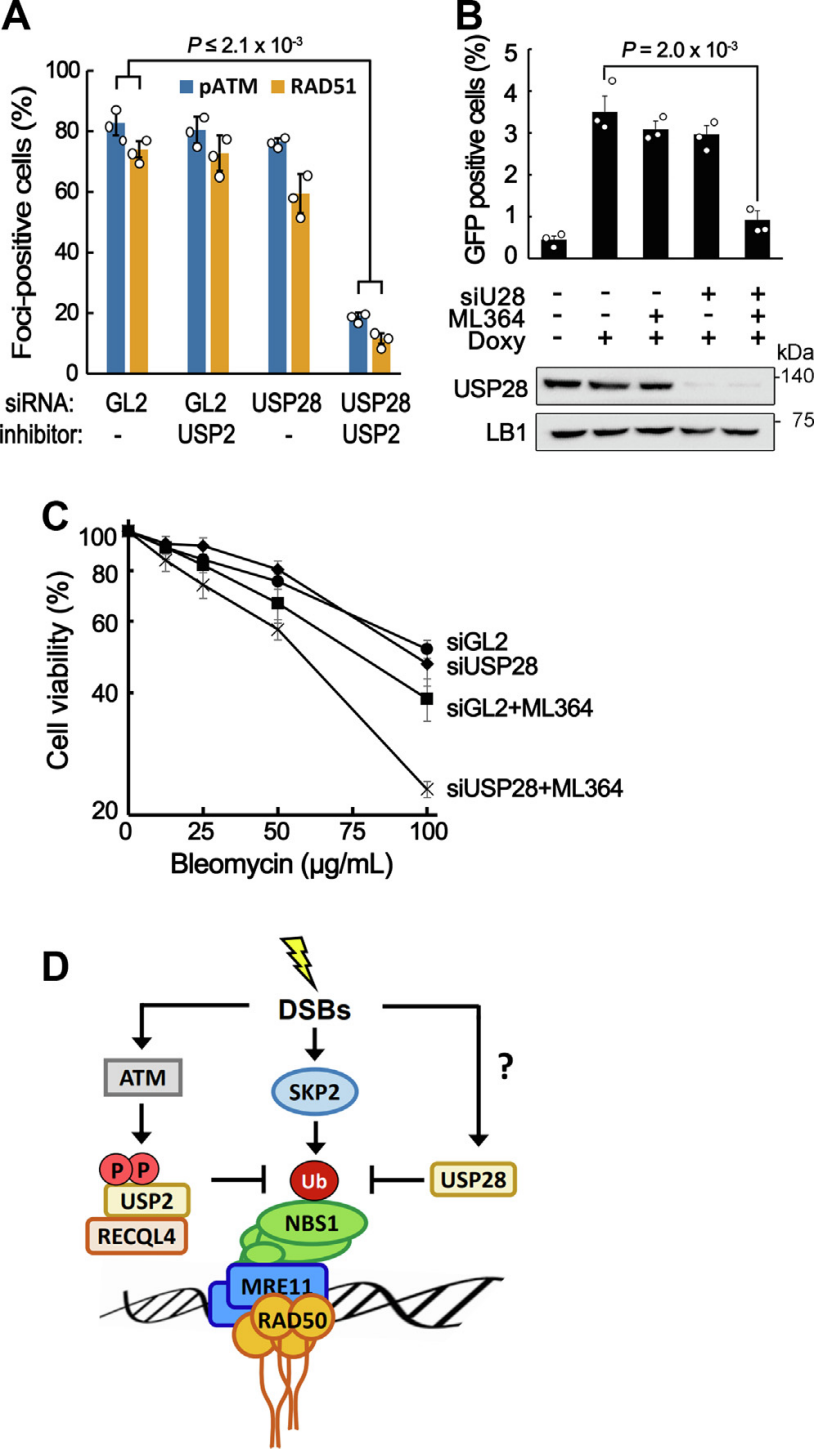
**Inactivation of both USP2 and USP28 activities results in defects in ATM activation and HR repair abilities.***A*, percentages of pATM or RAD51 foci-positive cells after NCS treatment in USP28 depleted and/or USP2 inhibited U2OS cells. Data in graphs are means ± SD; n = 3. *p* values for pATM, 2.1 × 10^−3^; RAD51, 1.2 × 10^−3^. *B*, HR repair assay using TRI-DR-U2OS cells. Cells transfected with the indicated siRNAs and incubated for 48 h in the presence or absence of ML164 (20 μM) after induction of I-*Sce*1 by doxycycline treatment. Data in graphs are means ± SD; n = 3. *Lower panel* is the Western blot showing the depletion of USP28. *C*, cell viability against bleomycin in USP28-depleted and/or USP2-inhibited cells. Cells were treated with various concentrations of bleomycin for 48 h, and WST-1 assay was carried out to measure the percentage of viable cells relative to undamaged cells. Data in graphs are means ± SEM; n = 3. *D*, diagram showing the control of NBS1 ubiquitination during the DSB response. DSB, double-strand break; HR, homologous recombination; NCS, neocarzinostatin; P, phosphorylation; Ub, ubiquitin.

Previously, USP28 was also shown to counteract NBS1 ubiquitination and prevent premature disassembly of the MRN complex from DSB sites in RECQL4-defective cells (24), and depletion of USP28 was shown to hardly influence the DSB response in many human cells, including U2OS (25). As both USP2 and USP28 can oppose NBS1 ubiquitination, we hypothesized that these two deubiquitinases play a redundant role in preventing premature disassembly of the MRN complex during the DSB response. To test this possibility, we examined the stability of the MRN complex at DSB sites in cells lacking the activity of both USP2 and USP28. EGFP-MRE11 proteins rapidly bound to the microirradiation site and the level of binding peaked at approximately 100 s (Fig. 6*A*). The level of EGFP-MRE11 proteins at the microirradiation site gradually decreased in cells treated with both USP2 inhibitor and USP28 siRNA, whereas the level of binding was stably maintained in mock- or single-treated cells (Fig. 6*A*). Early removal of the MRN complex from DSB sites was also confirmed by immunostaining of MRE11 foci after NCS treatment in cells treated with both USP2 inhibitor and USP28 siRNA (Fig. 6, *B* and *C*). An increase in the NBS1 ubiquitination and a decrease in the NBS1 stability were also observed after NCS treatment in cells inactivated with both USP2 and USP28 (Fig. 6, *D* and *E*), although a small decrease in the stability of NBS1 was also observed in the absence of DNA damage (Fig. 6*E* mock). Consistent with these observations, defects in ATM activation and HR repair abilities were observed only in cells inactivated with both USP2 and USP28 activities, whereas single inactivation of USP2 or USP28 only slightly decreased these abilities (Fig. 7, *A* and *B*). Sensitivity to the DSB-inducing reagent, bleomycin, also increased mostly by the inactivation of both USP2 and USP28 deubiquitinases (Fig. 7*C*). Taken together, these results strongly suggest that USP2 and USP28 play a redundant role in the DSB response to stably maintain the MRN complex on DSB sites by counteracting the ubiquitination of NBS1, and their role is essential for the proper cellular response to DSBs.

## Discussion

Various ubiquitin ligases and deubiquitinases are intertwined at the DSB site to control the recruitment, molecular interactions, and dissociation of factors involved in the DSB response in mammalian cells (30). In this study, we identified a deubiquitinase, USP2, as a new player acting at the DSB site by opposing the ubiquitination of NBS1, thereby preventing premature disassembly of the MRN complex from DSB sites.

USP2 is known to be involved in many cellular processes, including circadian rhythm, cell cycle, and cell proliferation (31, 32). USP2 is strongly associated with cell proliferation control and carcinogenesis by targeting proteins such as MDM2 (33), cyclin D1 (34), cyclin A1 (35), Aurora-A (36), and β-catenin (37). However, its involvement in the DSB response has not been shown before, at least partly due to the presence of another deubiquitinase, USP28, which plays a redundant role with USP2, as shown in this study. USP28 is known to be involved in cancer-related pathways by antagonizing FBW7-dependent ubiquitination of several proteins, such as C-MYC, C-JUN, ΔNp63, and Hif-1α (38, 39, 40). Initially, USP28 was reported to play an important role in the DSB response because depletion of USP28 results in the reduction of protein levels of 53BP1, CHK2, MDC1, and NBS1 after ionizing radiation in H460 lung carcinoma cells (25). The recruitment of USP28 proteins to DSB sites through interaction with the tandem BRCT domains of 53BP1 also supports this notion (41). However, the association of USP28 with the DSB response has been controversial because depletion of USP28 did not influence the stability of these proteins or the repair ability in other cell lines, including U2OS cells (25, 41). By inactivating both USP2 and USP28 simultaneously in this study, we clearly showed that these two deubiquitinases play a redundant role in counteracting NBS1 ubiquitination in the DSB response and that their activity is critical for proper cellular response to DSBs (Figs. 6 and 7). Therefore, the decrease in NBS1 protein levels in USP28-depleted H460 cells might be caused by defects in or reduced activity of other players acting in the control of NBS1. Consistent with this notion, we found in the Cancer Cell Line Encyclopedia gene expression database that the expression level of USP2 was much lower in H460 cells than in U2OS or HeLa cells (Table S1).

As shown in this study, USP2 stably interacts with NBS1 (Fig. 2) and affects ubiquitination of NBS1 (Figs. 2 and 6). Therefore, USP2 appears to act directly to reduce NBS1 ubiquitination and to stabilize the MRN complex. However, it may also be possible that USP2 acts indirectly to stabilize NBS1. Recently, USP2 was shown to interact with SKP2 *via* its substrate-binding domain of SKP2 and to stabilize substrates of SKP2-SCF E3 ligase, such as p21, independently of its deubiquitinase activity (42). As SKP2 E3 ligase is responsible for NBS1 ubiquitination during the DSB response, USP2 may also be able to reduce NBS1 ubiquitination through interaction with SKP2, although the importance of this mode of action in stabilizing the MRN complex during the DSB response has not yet been determined.

The MRN complex is one of the key factors in the DSB response, and its recruitment to DSB sites and its roles in the DSB response have been well documented in many previous studies. However, the mechanism of its removal from DSB sites is not clearly understood. Previously, K48-linked ubiquitination of the NBS1 protein by SKP2 E3 ligase was shown to increase after DSBs and to be responsible for premature disassembly of the MRN complex from DSB sites in cells lacking RECQL4 helicase activity (24). However, its role and control in cells with intact RecQL4 helicase activity remain unknown. In this study, we found that defects in deubiquitinases counteracting the NBS1 ubiquitination resulted in the premature disassembly of the MRN complex from DSB sites in cells with intact RECQL4 helicase activity (Figs. 2 and 6). Based on this observation, we suggest that K48-linked ubiquitination of the NBS1 protein by SKP2 E3 ligase is responsible for the removal of the MRN complex from DSB sites and opposing activity by deubiquitinases plays a critical role in determining the timing of its removal. The suggested mechanism controlling the stability of the MRN complex at DSB sites is summarized in Figure 7*D*. Although we do not clearly understand the control mechanism of USP28, ATM-dependent control of USP2 recruitment to DSB sites (Figure 3, Figure 4, Figure 5) suggested that USP2 plays a role in reducing NBS1 ubiquitination and stabilizing the MRN complex only when ATM kinase activity is maintained. Therefore, repair of DSBs and concomitant decrease in ATM activity may result in the dissociation of deubiquitinases and ubiquitination-dependent removal of the MRN complex from DSB sites. Interestingly, in this control of NBS1 ubiquitination, DSBs increase both ubiquitination activity and opposing activity simultaneously at DSB sites. In many ubiquitination-dependent controls of protein stability, timely activation of E3 ubiquitin ligase is a critical step (43, 44). However, activation of SKP2 E3 ligase for NBS1 ubiquitination appears to occur before its target is removed because the MRN complex should be stably maintained until the repair is completed. As SKP2 also plays a role in activating ATM by K63-linked ubiquitination of NBS1 (22), early recruitment of SKP2 to DSB sites seems inevitable. Therefore, the recruitment of deubiquitinases to prevent premature disassembly of the MRN complex is also required. Furthermore, excessive accumulation of the MRN complex, which may result in genome instability, can be effectively prevented by activating E3 ligase activity to remove the MRN complex at the beginning of the DSB response. Because MRE11 has nuclease activity (45), failure to remove the MRN complex can result in repair defects by excessive MRE11-mediated end resection (46). In addition, uncontrolled or enhanced HR activity has been shown to increase genome instability (47, 48). Therefore, complete and timely removal of the MRN complex is important for genome integrity and control of continued ubiquitinating activity by deubiquitinases as long as ATM is active appears to be an efficient strategy to prevent unwanted accumulation of the MRN complex after DNA damage.

The p97/VCP, which unfolds or segregates ubiquitinated substrates from their partners, is an important component of the ubiquitin-proteasome system (49), and association of the p97/VCP in the DSB response has been demonstrated in a few studies. The p97/VCP was shown to be important for the removal of RNF8 from DSB sites and cell survival after ionizing radiation-induced genotoxic stress (50). Extraction of the KU 70/80 heterodimer trapped in DNA was also carried out by p97/VCP recognizing ubiquitinated KU 80 (51). Recently, inactivation of p97 was shown to reduce the disassembly of the MRN complex from DSB sites, resulting in defective DNA repair by excessive end-resection (46). While MRE11 was postulated as a target of p97/VCP in this study, ubiquitinated NBS1 at the DSB sites can also be a target of p97/VCP. To test this possibility, we examined whether inhibition of p97/VCP prevents premature disassembly of the MRN complex from DSB sites after inactivation of both USP2 and USP28. While treatment with the p97/VCP inhibitor, DBeQ, did not completely prevent premature disassembly of the MRN complex under these conditions, dissociation of the MRN complex from DSB sites was significantly reduced by inhibition of p97/VCP (Fig. S3). Therefore, dissociation of the MRN complex from DSB sites by NBS1 ubiquitination also, at least, partly requires p97/VCP activity.

Previously, RECQL4 was shown to play an essential role in the DSB response by preventing premature disassembly of the MRN complex from DSB sites (24). As shown in Figure 3, Figure 4, Figure 5, USP2 stably interacted with RECQL4 in an ATM-dependent manner and recruitment of USP2 to DSB sites also requires RECQL4. Therefore, the essential role of RECQL4 in the DSB response appears to be mediated by deubiquitinases that counteract NBS1 ubiquitination, such as USP2. Meanwhile, the essential role of RECQL4 in ATM-dependent recruitment of USP2 to DSB sites raises questions about how overexpressed USP2 stabilizes the MRN complex in the absence of RECQL4 as shown in Figure 1. In RECQL4-depleted cells, USP2 cannot be recruited to DSB sites through ATM-dependent interaction with RECQL4 proteins. However, USP2 can interact with NBS1 protein when both proteins are overexpressed (Fig. 2*C*), and direct interaction between USP2 and SKP2 was also observed in a previous study (42). Therefore, USP2 has a potential to directly influence the MRN stability either by deubiquitinating NBS1 or inhibiting SKP2 E3 ligase in the absence of RECQL4. An increase in USP2 proteins due to overexpression may reinforce these modes of action that are not effective in normal circumstances. Interestingly, the helicase-defective mutant (Walker B mutant) of RECQL4 still supports the recruitment of USP2 to DSB sites (Fig. 3), which appears to contradict previous observations that support the essential role of RECQL4 helicase activity in the DSB response (24, 52, 53, 54, 55). While the initial recruitment of USP2 appeared to be normal in cells with RECQL4 lacking helicase activity, dissociation of USP2 from DSB sites was much faster in these cells than in cells with WT RECQL4 (Fig. 3*D*). Therefore, RECQL4 appears to play an additional role in stabilizing USP2 at the DSB sites, which requires helicase activity. The helicase activity of RECQL4 has the potential to influence the binding or removal of proteins by affecting the DNA or chromatin structures around DSB sites. However, it remains unclear how the helicase activity of RECQL4 contributes to the binding and maintenance of repair proteins at DSB sites.

## Experimental procedures

### Cell culture, DNA transfection, knockdown RNA transfection, and reagents

U2OS and HEK293T cells or TRI-DR-U2OS cells (56) were cultured in Dulbecco′s modified Eagle′s medium (Welgene), supplemented with 10% fetal bovine serum (FBS) (Welgene) or tetracycline-free FBS (Takara) and antibiotics (Welgene, Korea). Primary RTS skin fibroblast cells (AG17524 and AG18371) were purchased from Coriell Cell Repositories and cultured in alpha minimum essential medium Eagle (Welgene), supplemented with 15% FBS and antibiotics. All cells were maintained in a humidified incubator containing 5% CO_2_ at 37 °C. Transfection of DNA plasmids was performed with PolyFect (QIAGEN) or Lipofectamine 3000 (Invitrogen). For protein depletion, siRNAs were transfected using a Neon electroporator (Invitrogen) and incubated for 48 h. All siRNAs were synthesized by Bioneer. The sequences of the sense strand of siRNAs used in this study were as follows: GL2 (targeting firefly luciferase), 5′-AACGUACGCGGAAUACUUCGA-3′; RECQL4, 5′-GACUGAGGACCUGGGCAAA-3′; USP28, 5′- CUGCAUUCACCUUAUCAUU-3′. To induce DNA DSBs, the cells were treated with NCS (N9162, Sigma–Aldrich) or bleomycin (sc-200134, Santa Cruz). The proteasome inhibitor, MG132, was purchased from Apexbio (A2585). The USP2 inhibitor, ML364, was from Axon Medchem (2678). The ATM inhibitor, KU-55933 (SML1109), was from Sigma–Aldrich. The DNA-PK inhibitor, NU7441 (Axon 1463), was from Axon Medchem (Axon2678). The PARP inhibitor, olaparib, was from Selleckchem and the PARG inhibitor, PDD00017273, was from Sigma–Aldrich.

### Plasmids preparation

Amino acid substitutions in the RECQL4 Walker B motif (D605A and E606A) were generated by PCR and subcloned into the pcDNA3.1 (-) plasmid. For WT or various mutant USP2 protein expression, WT USP2 complementary DNA, N-terminal region complementary DNA (1–160 aa for CD1, 1–258 aa for CD2), and C terminal region (350–605 aa for ND1, 250–605 aa for ND2) were amplified by PCR and inserted into the pcDNA3.1 (-) plasmids containing various epitope tags. For alanine or glutamic acid substitution of the ATM phosphorylation sites of USP2 (S2, S96, T137, S142), a site-directed mutagenesis kit from Enzynomics (EZ004S) was used according to the manufacturer’s instructions.

### Antibodies

The primary antibodies used in this study were as follows: anti-RECQL4 antibody was prepared as previously described (24). Other antibodies used in this study included anti-pATM (Ser-1981, #4526, Cell Signaling), anti-MRE11 (GTX30294, GeneTex), anti-NBS1 (A7703, ABclonal), anti-RAD51 (GTX100469, GeneTex), anti-USP2 (A10399, ABclonal), anti-USP28 (A9292, ABclonal), anti-γH2AX (A300-081A, Bethyl and 05-636, EMD Millipore), anti-Lamin B1 (ab16048, Abcam), anti-GFP (sc-9996, Santa Cruz), anti-FLAG M2 (Sigma), and anti-HA (AE008, ABclonal). Alexa Fluor 488 antimouse IgG (A11001, ThermoFisher), Alexa Fluor 488 anti-rabbit IgG (A11008, ThermoFisher), Alexa Fluor 594 antimouse IgG (A11005, ThermoFisher), and Alexa Fluor 594 anti-rabbit IgG (A11012, ThermoFisher) were used as secondary antibodies for immunofluorescence microscopy.

### Immunofluorescence staining

For immunostaining of MRE11, NBS1, RAD51, and pATM, U2OS cells grown on coverslips were treated with 200 ng/ml NCS for 15 min and incubated in fresh medium for the indicated time. Cells were then pretreated with a cold buffer containing nonionic detergent (10 mM Pipes pH 7.0, 100 mM NaCl, 300 mM sucrose, 3 mM MgCl_2_, 0.5% Triton X-100) on ice for 5 min. Subsequently, cells were fixed with 3.7% paraformaldehyde in PBS for 10 min at 25 °C. The fixed cells were washed with 0.25% Triton X-100 in PBS on ice for 10 min and incubated in a blocking buffer (5% bovine serum albumin and 0.1% Tween-20 in PBS) for 30 min at 25 °C. Primary and fluorescent-conjugated secondary antibodies in the blocking buffer were subsequently added to the fixed cells for 1 h at 25 °C. The nuclei of cells were stained with 4′-6′-diamidino-2-phenylindole in mounting solution (VECTASHIELD, H-1200, Vector). Fluorescent images were obtained by fluorescence microscopy (Nikon TE2000-U or Zeiss LSM880), and cells containing 20 or more foci were counted as foci-positive cells for quantitation.

### Homologous recombination repair assay

The HR reporter cell line TRI-DR-U2OS carrying doxycycline-inducible I-*Sce*1 was obtained from Dr Philipp Oberdoerffer (NCI/NIH). Cells were treated with 10 μg/ml doxycycline for 48 h for I-*Sce*1 induction, and GPF-positive cells were analyzed using a flow cytometer (BD, Accuri).

### Immunoprecipitation and immunoblotting

For immunoprecipitation, cells were lysed in a buffer containing 40 mM Tris–HCl (pH 7.5), 100 mM NaCl, 2.5 mM MgCl_2_, 1 mM DTT, 5% glycerol, 0.2% NP-40, 20 mM NaF, 0.1 mM sodium orthovanadate, and protease inhibitors. After sonication, cell lysate was treated with benzonase (90 units/ml) at 4 °C for 4 h. The lysate was cleared by centrifugation at 18,000*g* for 10 min and the supernatant was used for immunoprecipitation. To prepare whole-cell extracts for immunoblotting, cells were lysed in a buffer containing 40 mM Tris–HCl (pH 7.5), 150 mM NaCl, 1% NP-40, 1 mM EDTA, 0.25% sodium deoxycholate, 20 mM NaF, 0.1 mM sodium orthovanadate, and protease inhibitors. The lysate was sheared by sonication. Protein concentration was quantified using the Bradford assay.

### Ubiquitination assay

The ubiquitination assay for Nbs1 was performed as described by Choo and Zhang (57) with slight modifications. HEK293T cells were transfected with expression vectors for HA-tagged ubiquitin and the indicated proteins and incubated for 24 h. The cells were pretreated with MG132 (40 μM) for 1 h and then treated with 200 ng/ml NCS for 1 h. Protein extracts were prepared by boiling the cells at 95 °C in a cell lysis buffer (20 mM Tris–HCl, pH 8.0, 150 mM NaCl, 2% SDS, 10 mM N-ethylmaleimide, and protease inhibitors) for 10 min, followed by shearing by sonication. The extracts were diluted nine times the volume of dilution buffer (10 mM Tris–HCl, pH 8.0, 150 mM NaCl, 2 mM EDTA, and 1% Triton X-100) and incubated at 4 °C for 30 min on the rotation wheel. The cell extracts were centrifuged at 18,000*g* for 30 min for clearing, and the supernatant was used for immunoprecipitation. Beads were washed twice with immunoprecipitation buffer and three times with wash buffer (10 mM Tris–HCl, pH 8.0, 1 M NaCl, 1 mM EDTA, and 1% NP-40).

### Laser microirradiation and real-time imaging of fluorescent proteins

For laser microirradiation, U2OS cells grown on a dish with a thin glass bottom (SPL, 101350) were treated with 5 μg/ml Hoechst 33342 10 min before microirradiation. Cells were locally irradiated with a fixed laser wavelength (405 nm, 100% laser output at a scan speed of 32.77 μs/pixel with 1–3 iteration) using a LSM880 laser confocal microscope system with a temperature-controlled CO_2_ chamber (Zeiss). The Plan-Apochromat 63× oil objective lens was used to observe the time-lapse images, and the fluorescence intensities of irradiated areas relative to nonirradiated areas within the nucleus were measured using ZEISS ZEN 2.3 SP1 software (Zeiss).

### Protein stability assay

The U2OS cells were transfected with the indicated siRNAs and incubated for 48 h. The cells were pretreated with 50 μg/ml cycloheximide (01810, Sigma–Aldrich) for 1 h and then treated with NCS (200 ng/ml). The cells were harvested and lysed at the indicated times, and the protein levels of NBS1 were quantified using a chemiluminescence imaging system (ATTO Ez-CaptureII, CS analyzer) after immunoblotting.

### WST-1 assay for cell viability

The assay was performed according to the manufacturer’s instructions (EZ-Cytox, DoGenBio). Briefly, U2OS cells were transfected with the indicated siRNAs and cultured in 96-well plates for 48 h. Cells were treated with different concentrations of bleomycin (sc-200134a, Santa Cruz) in the presence or absence of ML364. After 24 h of incubation, cells were treated with 10% WST-1 reagent (EZ-Cytox, DoGEN) for 30 min, and absorbance was measured using Epoch2 Microplate Spectrophotometer (BioTek).

### Statistics analysis

Statistical significance between groups was determined by two-tailed Student’s *t* test using GraphPad Prism5 software (GraphPad Software Inc). Data are presented as mean ± SD or SEM. All statistical tests are indicated in the figure legends.

## Data availability

All data are contained within the article.

## Supporting information

This article contains supporting information.

## Conflict of interest

The authors declare that they have no conflicts of interest with the contents of this article.
