# Supplementary material for: Deubiquitinase USP2 stabilizes the MRE11–RAD50–NBS1 complex at DNA double-strand break sites by counteracting the ubiquitination of NBS1
Source: J Biol Chem. 2022 Nov 25;299(1):102752. doi: 10.1016/j.jbc.2022.102752 (PMC9758435; doi:10.1016/j.jbc.2022.102752)
Supplement: Supplemental Figures S1–S3 and Table S1 [file mmc1.docx]

**Supplementary information**

Deubiquitinase Usp2 stabilizes the MRE11-RAD50-NBS1 complex at DNA double-strand break sites by counteracting the ubiquitination of NBS1

Hyunsup Kim^1^, Dongmin Kim^2^, Hyemin Choi^2^, Gwangsu Shin^1^, and Joon-Kyu Lee^1,2*^

Table S1, Figure S1, Figure S2, Figure S3

| cell line | Depmap ID | USP2 (9099) | USP28 (57646) |
| --- | --- | --- | --- |
| U2OS | ACH-000364 | 1.86393845 | 4.993674362 |
| HELA | ACH-001086 | 2.266036894 | 3.40599236 |
| NCI-H460 | ACH-000463 | 0.650764559 | 4.175524601 |

**Table S1.** Relative expression levels of Usp2 and Usp28 in U2OS, HELA, or H460 cell lines found in Cancer Cell Line Encyclopedia.


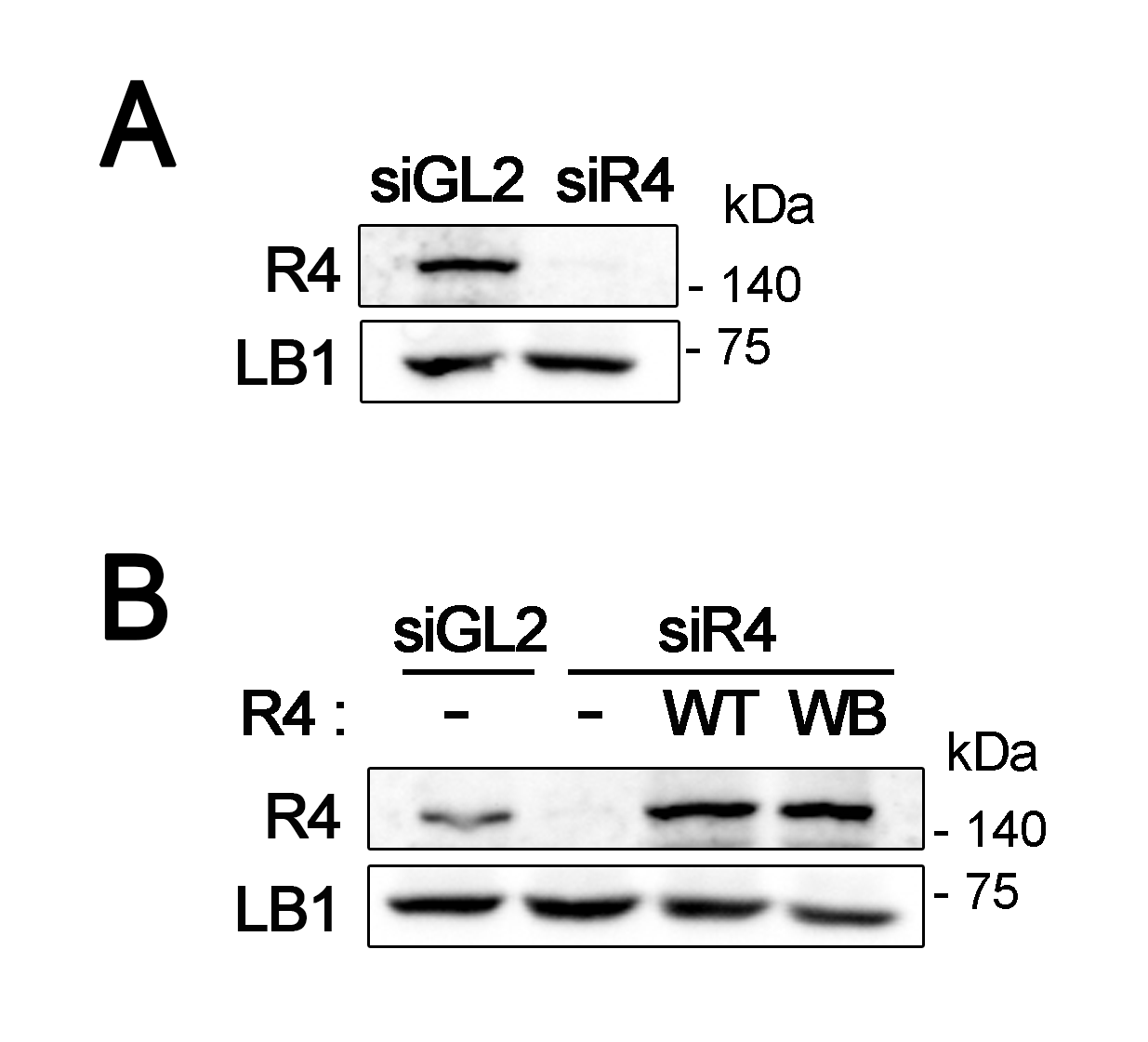


**Figure S1. RECQL4 protein levels in U2OS cells transfected with RECQL4 siRNAs or RECQL4 expression plasmids.** *A,* Western blot of U2OS cells used in Fig. 3C. U2OS cells were transfected with RECQL4 siRNA and incubated for 48 h. *B,* Western blot of cells used in Fig. 3D. U2OS cells transfected with RECQL4 siRNA were transfected with wild type (WT) or walker B mutant (WB) RECQL4 plasmids. LB1, lamin B1; R4, RECQL4.


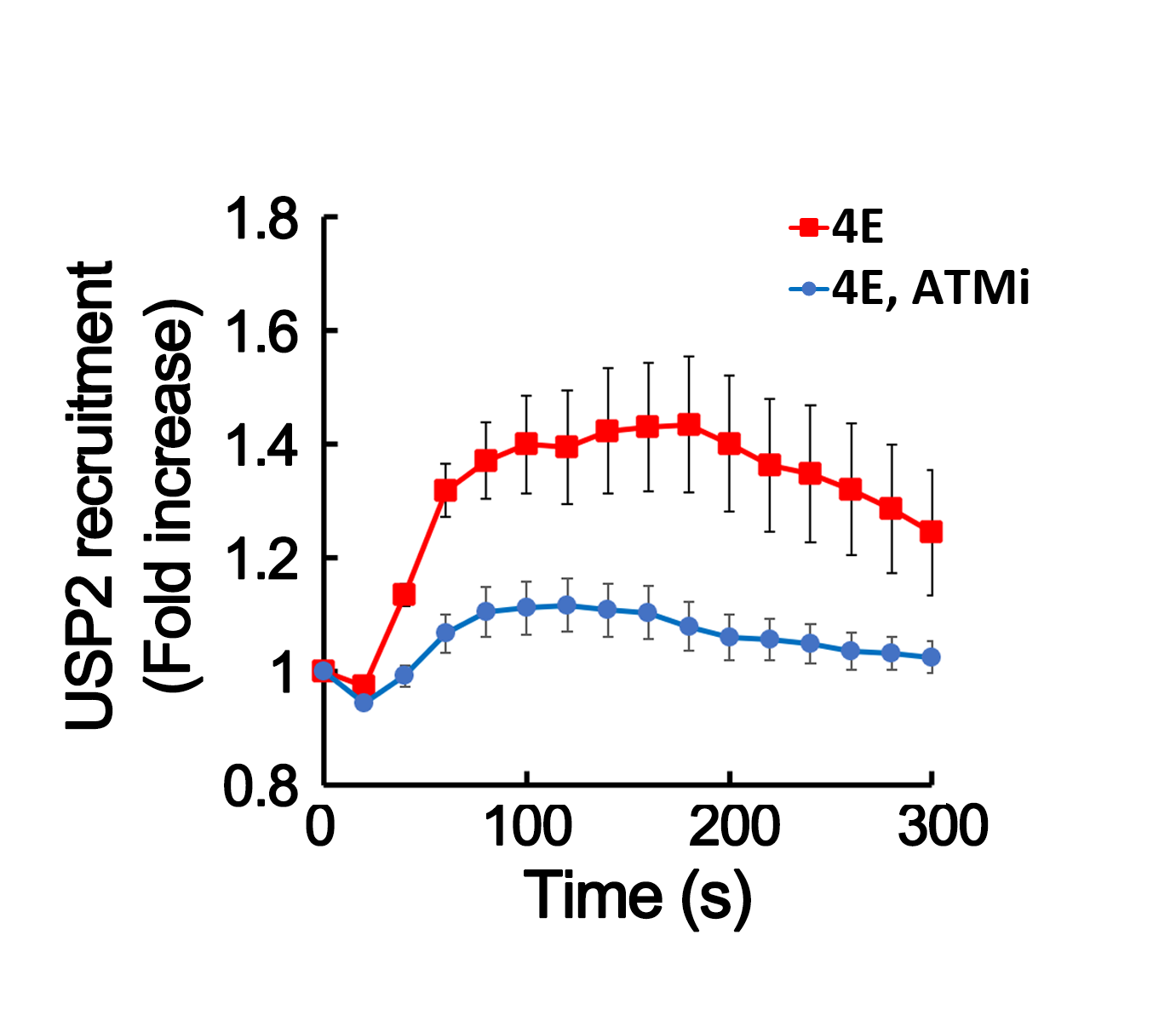


**Figure S2. Recruitment of phospho-mimetic mutant USP2 (4E) to DSB sites is prevented by inhibition of ATM.** Recruitment of EGFP-fused phospho-mimetic mutant USP2 (4E) was examined by laser micro-irradiation followed by live cell imaging. ATM inhibitor (ATMi), KU55933, was treated 1 h before micro-irradiation. 4E, USP2 with glutamic acid substitution of four putative ATM phosphorylation sites (S2, S94, T137, S142) in the N-terminus. Data in graphs are means ± SEM; n =20.


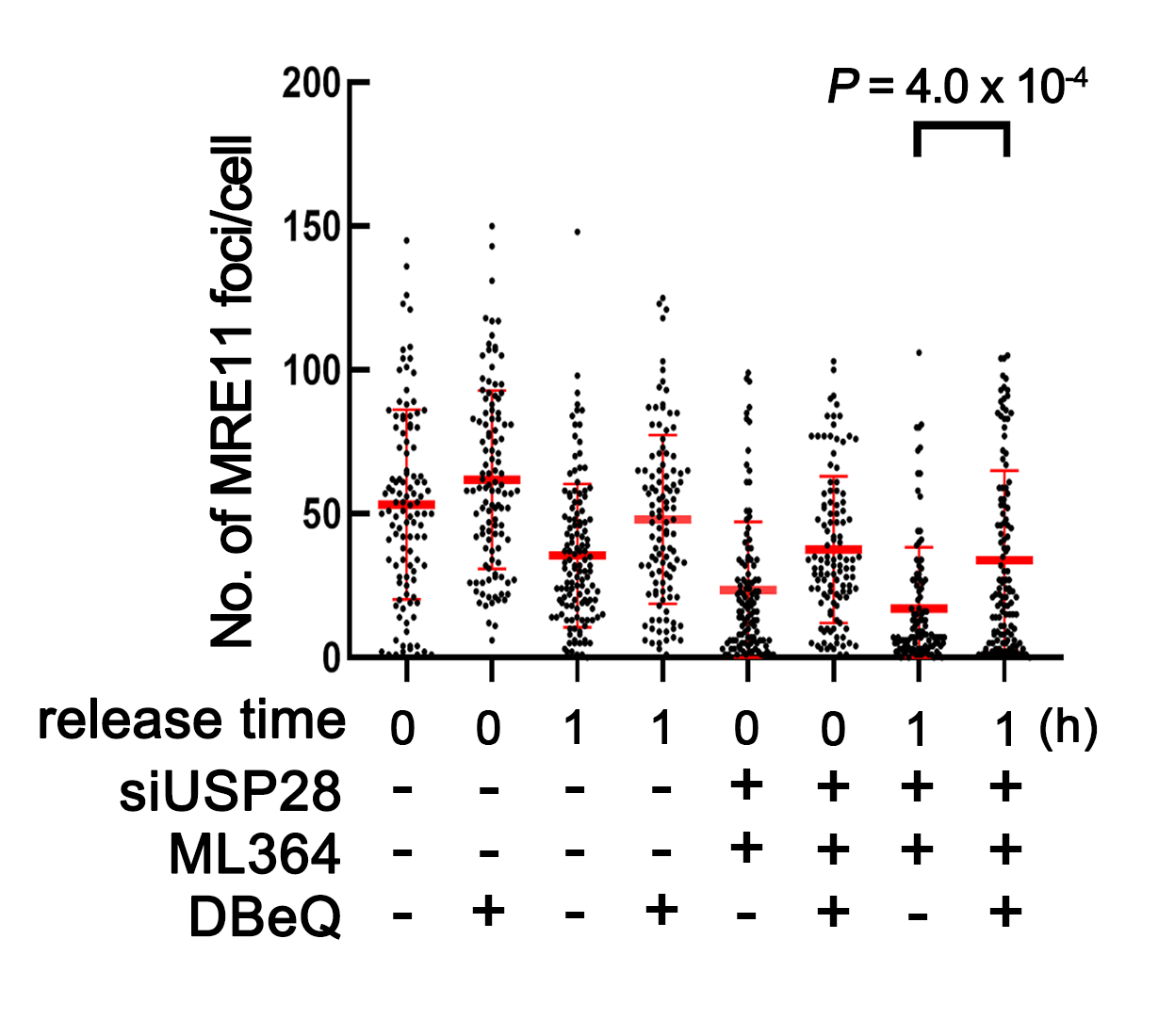


**Figure S3. Inhibition of p97/VCP prevents dissociation of the MRN complex from DSB sites.** U2OS cells transfected with indicated siRNAs and treated with inhibitors as indicated were treated with NCS for 15 min and incubated in a fresh medium for 0 h and 1 h. MRE11 immunofluorescence staining was performed. For quantitation, 100 cells were counted and number of foci per cell were shown. Data in graphs are means ± SD.
